# circCOL1A1 Promotes the Progression of Gastric Cancer Cells through Sponging miR-145 to Enhance RABL3 Expression

**DOI:** 10.1155/2021/6724854

**Published:** 2021-09-29

**Authors:** Yue Ma, Yanyi Ren, Huitao Wen, Chengcheng Cui

**Affiliations:** ^1^Department of Gastroenterology, Hospital of Chengdu University of Traditional Chinese Medicine, No. 39 Shi-er-qiao Road, Chengdu 610072, Sichuan Province, China; ^2^Department of Nephrology, Chengdu First People's Hospital, No. 18 Norn Vientiane Road, Chengdu, Hi-Tech Zone 610041, Sichuan Province, China; ^3^Department of Pediatrics, Huai'an Second People's Hospital, The Affiliated Huai'an Hospital of Xuzhou Medical University, Huai'an, China

## Abstract

Circular RNA has been reported to be a new noncoding RNA which plays important roles in tumor progression. One of the most common functions of circular RNA is to regulate microRNA expression by acting as a microRNA sponge. However, the circular RNA expression profile and function remain mostly unclear in gastric cancer. In the study, we explored the expression and function of circCOL1A1 (hsa_circ_0044556) in gastric cancer. We performed RT-PCR with divergent primers, mRNA stability assay, and RNase R digestion assay to characterize circCOL1A1 in gastric cancer cell lines. qRT-PCR was applied to detect the level of circCOL1A1 in both gastric cancer cell lines and tissues. Gain- and loss-of-function studies were carried out to detect the influence of circCOL1A1 on gastric cancer cells by performing CCK8, migration, and invasion assays. The regulation of the downstream genes was identified by qRT-PCR, western blot assay, dual luciferase assay, and RNA pull-down assay. The results showed that circCOL1A1 was highly expressed in both gastric cancer cells and tissues. Silence of circCOL1A1 inhibited the proliferation, migration, and invasion of gastric cancer cells. circCOL1A1 regulated the expression of miR-145 by acting as a microRNA sponge, and the influence of circCOL1A1 could be abrogated by miR-145 mimics. Our research shows that miR-145 plays its functions through targeting and regulating RABL3. Inhibition of circCOL1A1/miR-145/RABL3 could effectively suppress gastric cancer cell proliferation, migration, and invasion. circCOL1A1 also promote the transformation of M1 into M2 macrophage. Our study identified circCOL1A1 as a novel oncogenic circRNA and will provide more information for gastric cancer therapy.

## 1. Introduction

### 1.1. Cell Culture

MKN-45, SGC-7901, AGS, MGC-803, and BGC-823 gastric cancer cell lines were obtained from ATCC (American Type Culture Collection). Gastric cancer cell lines were cultured in RPMI 1640 with 10% FBS. GES-1 is a normal gastric epithelial cell and obtained from Cell Resource Center of China. GES-1 was cultured in DMEM with 10% FBS.

### 1.2. Clinical Cancer Tissues

20 gastric cancer and adjacent tissues were obtained from Huai'an Second People's Hospital. The Huai'an Second People's Hospital ethics committee approved the usage of the patients' tissues. All experiments followed the guidelines of the ethic committee. All patients have been informed about the usage of the tissue and consented to the study. All tissues were stored at -80°C.

### 1.3. Construction of Plasmids and Transfections

The sequence of circCOL1A1 was synthesized and inserted into the PGL3 plasmid by Geenseed Biotech. siRNAs targeting circCOL1A1, miR-145 inhibitor, and miR-145 mimics were obtained from GenePharma (Shanghai, China). All gastric cells were transfected with Lippo 3000 under the direction of the manufactory.

### 1.4. Cell Proliferation Assay

Cell proliferation was detected by using the CCK8 kit (Dojindo Molecular Technologies, Kumamoto, Japan) following the instruction. 4000 cells were seeded per well in 96 well plates.10 *μ*l CCK8 solution was added per well. We detected the absorbance at 450 nm 2 hours later.

### 1.5. Migration Assay

We applied Transwell chambers (Corning, NY, USA) to detect cancer cell migration. 2.5 × 10^4^ gastric cells were suspended in 1640 medium and then plated in the upper chamber. 800 *μ*l 1640 with 20% FBS was added to the lower chamber. 48 hours later, cells on the membrane were fixed with 4% PFA and dyed with 2% crystal violet. The migration cells were imaged and counted under 20x magnification.

### 1.6. Invasion Assay

We applied Transwell chambers with 50 *μ*l Matrigel (Corning, NY, USA) to detect cancer cell invasion. 5 × 10^4^ gastric cells were suspended in 1640 medium and then plated in the upper chamber. 800 *μ*l 1640 with 20% FBS was added to the lower chamber. 48 hours later, cells on the membrane were fixed with 4% PFA and dyed with 2% crystal violet. The migration cells were imaged and counted under 20x magnification.

### 1.7. Quantitative Real-Time PCR

We isolated RNA using Roche Isolation Reagent following the protocol. cDNA was synthesized by using the MMLV RT Kit (ABI, Warrington, UK). Quantitative real-time PCR (qRT-PCR) was performed with SYBR PCR Kit (Roche, Basel, Switzerland). PCR was performed in ABI 7500 System. Relative expression of the gene was calculated with the *ΔΔ*Ct method. We used GAPDH as the control gene. The primers are shown in Table 1.

### 1.8. Western Blot

We used RIPA buffer to extract proteins from gastric cancer cells. Western blot was carried out as the previous study showed [1]. Antibody for RABL3 (PA5-57178) and antibody for *β*-actin (MA1-744) were obtained from Thermo Fisher.

### 1.9. Transfections

The gastric cancer cells were seeded in cell culture plates of 6 wells at 37°C. circCOL1A1 siRNA, circCOL1A1 overexpression plasmids, miRNA inhibitor, and miRNA mimics were brought from GenePharma (Shanghai, China) and transfected into gastric cells by Lipofectamine 2000 (Invitrogen, Carlsbad, CA, USA) under the direction of the manufactory.

### 1.10. Dual-Luciferase Reporter Assay

We inserted the sequence of circCOL1A1 into PGL3 vector (Promega, WI, USA). Cells were cultured in 24-well plates and then transfected with the indicated plasmids. We applied dual-luciferase system (Promega) to detect the luciferase activity 48 hours after transfection [2].

### 1.11. Digestion of RNase R and mRNA Stability Assay

RNA was treated with RNase R at the concentration of 4 U/*μ*g for 30 min at 37°C. To detect the mRNA stability, we treated the RNA with actinomycin at the concentration of 6 mg/ml for 0 h, 4 h, 8 h, 12 h, and16 h. qRT-PCR was applied to measure the RNA level. GAPDH was selected as the control. All experiments were performed in triplicate.

### 1.12. Subcellular Fractionation Location

We extracted nuclear and cytoplasmic RNA using the Cytoplasmic and Nuclear RNA Purification Kit (Invitrogen) under the direction of protocol. qRT-PCR was carried out to detect cytoplasmic and nuclear circCOL1A1 levels. We used GAPDH as cytoplasm control and U2 as a nuclear control.

### 1.13. Flow Cytometry

AGS or MKN-45 cells were cultured with M0-type macrophage. We stained the macrophage with CD206 and CD86 (BD Biosciences Pharmingen, USA) 30 min under dark. The isotype antibody was also added. We used flow cytometry (BD, USA) to detect the expression of CD86 for M1 macrophages and CD206 for M2 macrophage.

### 1.14. RNA Pull-Down Assay

Biotin-labeled circCOL1A1 was incubated with streptavidin-labeled beads. Then, the gastric cancer cell lysis was treated with coated beads. We applied qRT-PCR to analyze the pull-down compounds [2].

### 1.15. Xenograft Tumor Assay

The animal experiments were carried out under the animal ARRIVE guidelines and have been approved by Xuzhou Medical University Animal Committee. The group allocation was blinded to the investigators. The nude mice were randomly divided into two groups (*n* = 6). 2 × 10^6^ AGS were subcutaneously injected into the upper black of 5-week-old nude mice. We measured the tumor growth every three days and calculated the volume as Volume = 1/2∗length∗width^2^.

### 1.16. Statistical Analysis

GraphPad Prism 5 (GraphPad Software, CA, USA) was used to analyze the data. All results were shown as mean ± SD. We used analysis of variance (ANOVA) and Student's *t*-test to measure the significance between the groups. The expression correlation was analyzed by Pearson's correlation. All analyses were two-sided. *P* < 0.05 was statistically significant.

## 2. Methods and Materials

Gastric cancer (GC) has been one of the most common cancers which caused a large number of tumor-related deaths [4]. Although gastric cancer therapy developed in the past decades, the five-year survival of gastric patients in most countries remains less than 28%. Most patients are diagnosed with advanced gastric cancer, and the median survival decreased to 8 to 11 months [5]. The poor prognosis stems from the lack of early diagnosis and effectively cancer therapy. Therefore, research focusing on the underlying mechanisms of gastric cancer progression is of great importance for gastric cancer therapy.

Circular RNA (circRNA) is a new class of endogenous noncoding RNA (ncRNA). The main difference between circular RNA and linear RNA is the special structure which is a closed loop without 5′ end cap or 3′ end poly A tail. The special structure is with resistance to the RNase R and contributes the stability of circular RNA. Most of the circular RNAs generate from exons and exist in the cytoplasm. Few exonic-intronic circRNAs and intronic circRNAs are found to exist in nuclear [6]. Circular RNAs are widely expressed in various cells with abundance, suggesting important functions in cell signaling. Circular RNAs have microRNA response elements (MRE) and act as a competing endogenous RNA (ceRNA) for microRNAs, which would strongly influence the microRNA expression and activity. Thus, acting as a sponge results in the regulation of the microRNA target gene. Increasingly circRNAs have been discovered in cancer tissues due to the development of RNA-sequencing technology. Emerging researches have shown that circRNAs play important functions in cancer progression through influencing tumor growth, migration invasion, and differentiation [3, 7].

Numerous researches have reported the function of circRNAs in gastric cancer [8, 9], hepatocellular carcinoma [10, 11], glioma [12], and lung cancer [13]. The roles of circRNA in gastric cancer remain in their infancy and need further study.

Various circRNAs were discovered to be expressed differently between gastric cancer tissue and adjacent tissues. Microarrays of circRNA were applied to search for the functional circRNAs [9]. circCOL1A1 (hsa_circ_0044556) generated from COL1A1 gene (Collagen, Type I, Alpha 1) and was located on chr17: 48271490-48272189. circCOL1A1 is 200 bp in length. COL1A1 has been reported to play important roles in disease progression including breast cancer [14], colorectal cancer [15], hepatocellular carcinoma [16], and oral squamous cell carcinoma [17]. However, the existence and function of circCOL1A1 have not been reported up to now. We performed both in vitro and in vivo experiments to figure out the function of circCOL1A1 in gastric cancer.

## 3. Results

### 3.1. circCOL1A1 Is Upregulated in Gastric Cancer Cells and Tissues

**Figure 1:**
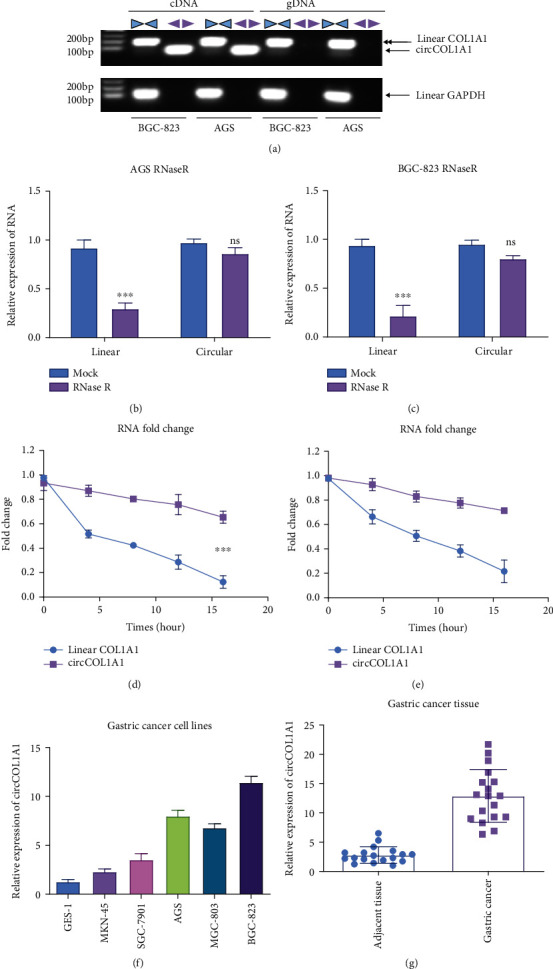
Characterization of circCOL1A1 in gastric cancer. (a) PCR was performed in BGC-823 and AGS. circCOL1A1 was amplified by specially designed divergent primers from cDNA but not from genomic DNA (gDNA). Linear COL1A1 RNA was amplified by convergent primers both in cDNA and gDNA. GAPDH was an endogenous control. (b, c) RNase R was used to pretreat the RNA, and qRT-PCR was used to determine the resistance. circCOL1A1 was resistance to RNase R in BGC-823 and AGS cell lines. (d, e) BGC-823 and AGS were treated with 5 mg/ml actinomycin D for 0, 4, 8, 12, and 16 h. RNAs extracted and determined by qRT-PCR analyses. (f) Expression of circCOL1A1 in different gastric cancer cells and normal cells. (g) qRT-PCR was applied to determine the expression of circCOL1A1 in gastric cancer tissue.

We performed numerous verification experiments to investigate the presence and function of circCOL1A1 in gastric cancer cells. We used divergent primers to amplify circCOL1A1 and convergent primers to amplify linear COL1A1 RNA. Results showed that the linear form of COL1A1 could be amplified from both cDNA and gDNA in BGC-823 and AGS gastric cancer cells, while circCOL1A1 could only be amplified in cDNA (Figure 1(a)). The biggest difference between circular RNAs and linear RNAs is that circular RNAs are resistance to RNase R. To confirm the features of circCOL1A1, we performed RNase R digestion assay. The results showed that circular COL1A1 was resistant to RNase R digestion, while linear COL1A1 was degraded by RNase R (Figures 1(b) and 1(c)). To detect the stability of linear RNA and circular RNA, we performed half-life experiments by using actinomycin D (ActD) to inhibit the transcription. Then, we carried out the qRT-PCR to monitor level of RNA. The results showed that circCOL1A1 exhibited high stability than its linear isoforms both in AGS (Figure 1(d)) and BGC-823 (Figure 1(e)). It has been reported that circular RNA could act as a microRNA sponge in the cytoplasm. The expression levels of circCOL1A1 were also tested in gastric cancer cell lines and clinical tissues. qRT-PCR results showed that circCOL1A1 was clearly upregulated both in gastric cancer cells (Figure 1(f)) and clinical gastric cancer tissues (Figures 1(g)). These results confirmed the existence and high expression of circCOL1A1 in gastric cancer

### 3.2. Overexpression of circCOL1A1 Enhances the Proliferation, Migration, and Invasion of Gastric Cancer Cells

**Figure 2:**
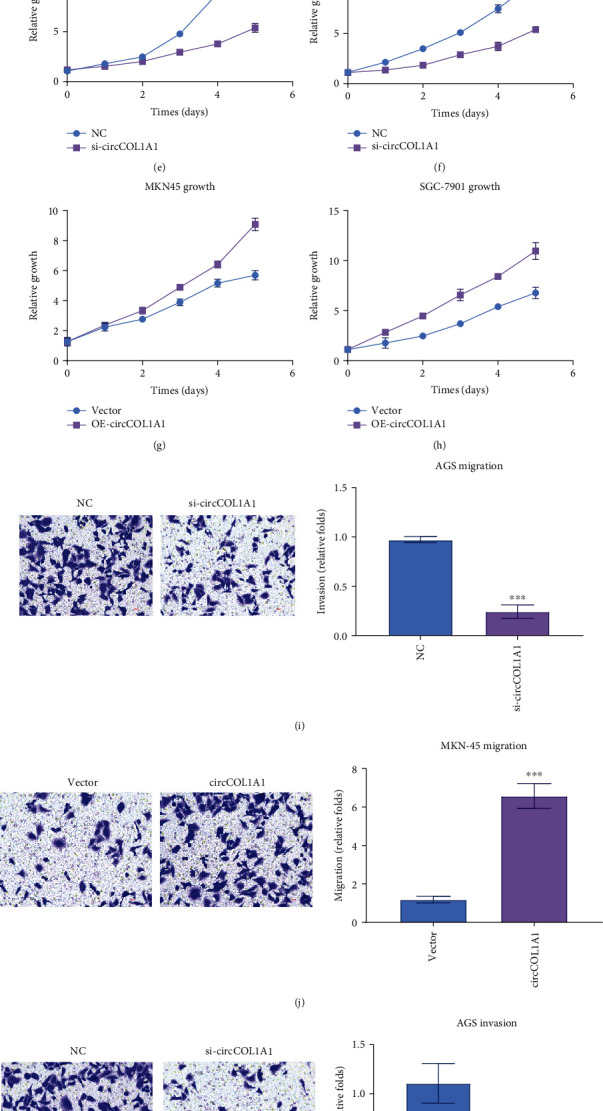
circCOL1A1 promotes the proliferation, migration, and invasion of gastric cancer cells. (a, b) Loss-of-function experiments were performed by transfecting siRNAs targeting circCOL1A1 into AGS and BGS-823 cancer cells. (c, d) Gain-of-function experiments were carried out by transfecting circCOL1A1 expression plasmids in MKN-45 and SGC-7901. (e–h) CCK8 assays were performed to detect the influence of circCOL1A1 on proliferation in gastric cancer cells. (i) Migration assays were performed to detect the influence of circCOL1A1 silence on AGS migration. (j) Migration assays were performed to detect the influence of circCOL1A1 overexpression on MKN-45 migration. (k) Invasion assays were performed to detect the influence of circCOL1A1 silence on AGS invasion. (l) Invasion assays were performed to detect the influence of circCOL1A1 overexpression on MKN-45 invasion.

We investigated the function of circCOL1A1 by gain- and loss-of-function experiments. We silenced circCOL1A1 in AGS and BGC-823 cells (Figures 2(a) and 2(b)) and overexpressed circCOL1A1 in MKN-45 and SGC-7901 (Figures 2(c) and 2(d)). We performed qRT-PCR to identify the level of circCOL1A1. We applied proliferation assay, migration assay, and invasion assay to investigate the function of circCOL1A1 in gastric cancer. We detect the proliferation of gastric cancer cell lines by CCK8 assay. The results showed that silencing of circCOL1A1 weakened the proliferation of AGS (Figure 2(e)) and BGC-823 (Figure 2(f)), and overexpression of circCOL1A1 enhanced the proliferation of MKN-45 (Figure 2(g)) and SGC-7901 (Figure 2(h)). What is more, the silence of circCOL1A1 also inhibited the migration and invasion of AGS (Figures 2(i) and 2(k)). Overexpression of circCOL1A1 significantly enhanced the migration and invasion abilities of MKN-45 (Figures 2(j) and 2(l)). Loss-of-function and gain-of-function experiments both confirmed that circCOL1A1 promoted the proliferation, migration, and invasion of gastric cancer cells.

### 3.3. circCOL1A1 Does Not Participate in the Regulation of COL1A1 mRNA and Protein

**Figure 3:**
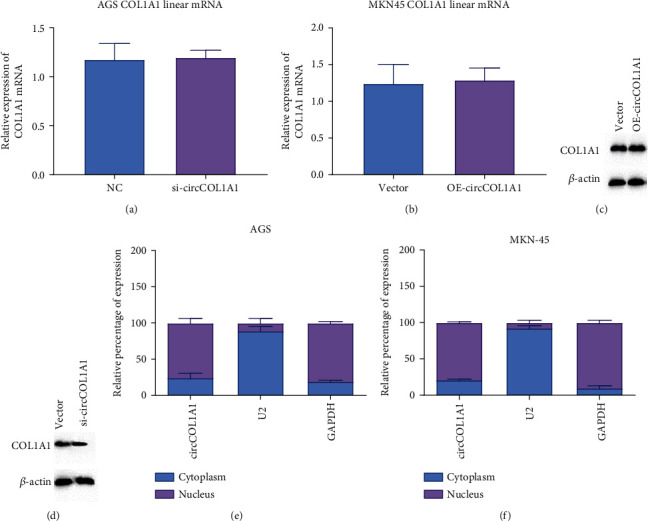
circCOL1A1 has no influence on COL1A1 protein level and mainly locates in the cytoplasm. (a) qRT-PCR was performed to detect COL1A1 linear RNA levels in AGS. (b) qRT-PCR was performed to detect COL1A1 linear RNA level in MKN-45. (c) Western blot was performed to detect COL1A1 protein levels in AGS. (d) Western blot was performed to detect COL1A1 protein levels in MKN-45. (e, f) Subcellular fractionation assay showed that circCOL1A1 was mainly located in the cytoplasm of AGS and MKN-45 cells. U2 and GAPDH were used as nuclear and cytoplasmic controls, respectively.

It has been reported that circular RNAs could participate in the splicing of pre-mRNA and regulate maturation and transcription of mRNA [18]. We first detected whether circCOL1A1 could influence COL1A1 mRNA transcription. The results showed that neither overexpression nor silence of circCOL1A1 influenced the mRNA and protein level of COL1A1 (Figures 3(a)–3(d)). The results confirm that circCOL1A1 does not participate in the regulation of COL1A1 mRNA and protein. As circular RNA could function as a sponge for microRNA and regulate the target gene of microRNA in the cytoplasm, we performed the subcellular fractionation assay and detected the location of circCOL1A1. Results showed that circCOL1A1 mainly existed in the cytoplasm (Figures 3(e) and 3(f)). All results above indicate the possible function of circCOL1A1 as a microRNA sponge.

### 3.4. circCOL1A1 Acts as a Sponge for miR-145

**Figure 4:**
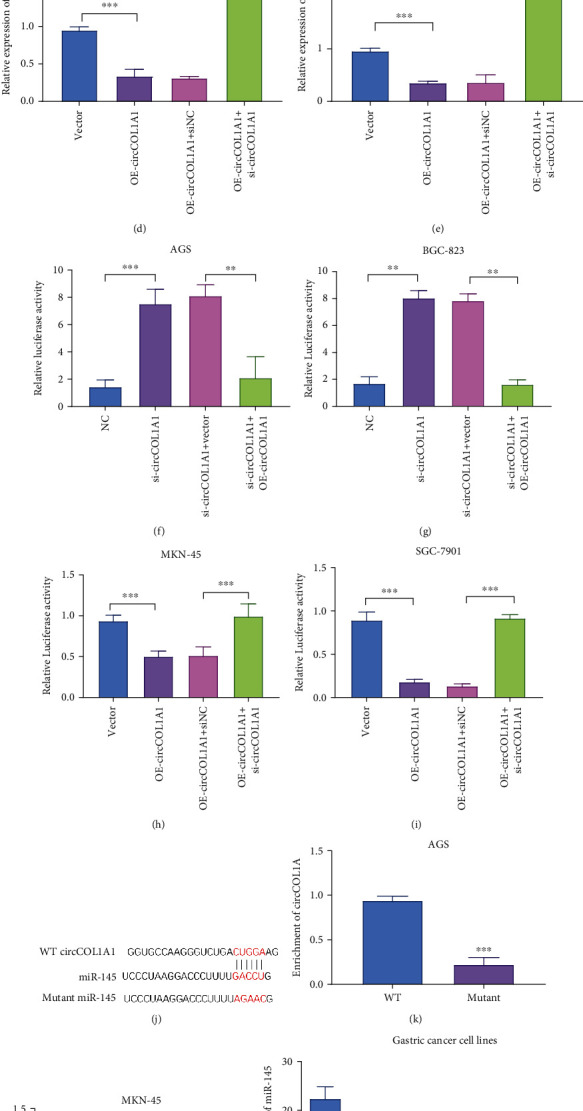
circCOL1A1 targets miR-145 by acting as a microRNA sponge. (a) Putative binding site between circCOL1A1 and miR-145 is shown. (b, c) circCOL1A1 was silenced in AGS and BGS-823 cells and reexpressed circCOL1A1 in AGS and BGS-823. qRT-PCR was performed. (d, e) circCOL1A1 was overexpressed in MKN-45 and SGC-7901 cells and then silenced circCOL1A1 in circCOL1A1 overexpressing cells. qRT-PCR was performed. (f, g) circCOL1A1 was silenced in AGS and BGS-823 cells and reexpressed circCOL1A1 in AGS and BGS-823. Luciferase reporter assays were performed. (h, i) circCOL1A1 was overexpressed in MKN-45 and SGC-7901 cells and then silenced circCOL1A1 in circCOL1A1 overexpressing cells. Luciferase reporter assays were performed. (j) The wild type and mutation of miR-145 were indicated. (k, l) RNA pull-down assays were performed to validate the direct interaction between miR-145 and circCOL1A1. (m) The expression level of miR-145 in gastric cancer cell lines by qRT-PCR. (n, o) The expression level of miR-145 in gastric cancer tissues by qRT-PCR.

As cytoplasmic circRNAs could act as a sponge for microRNA and inhibit the target genes of microRNA [19], we searched for the potential target of circCOL1A1 by Circular RNA Interactome (https://circinteractome.nia.nih.gov). We focus on miR-145, a tumor suppressor which is reported to play important roles in gastric cancers [20, 21].  Figure 4(a) showed the predicted binding sites between circCOL1A1 and miR-145. To validate the prediction, we performed the qRT-PCR after overexpressing and silencing of circCOL1A1 in gastric cells (Figures 4(b)–4(e)). qRT-PCR analyses showed that silencing of circCOL1A1 significantly increased the level of miR-145 and reoverexpression of circCOL1A1 effectively inhibited the expression of miR-145 in AGS (Figure 4(b)) and BGC-823 (Figure 4(c)). Also, overexpression of circCOL1A1 inhibited the expression of miR-145, and the rescue experiment could significantly upregulate the expression of miR-145 in MKN-45 (Figure 4(d)) and SGC-7901 (Figure 4(e)). Luciferase activity was also detected in the gain-of-function and loss-of-function experiments. Overexpression of circCOL1A1 enhanced the luciferase activity of miR-145 and silencing of circCOL1A1 inhibited the activity of miR-145 (Figures 4(f)–4(i)).

To confirm whether there was a direct interaction between circCOL1A1 and miR-145, we performed RNA pull-down in AGS (Figure 4(k)) and MKN-45 (Figure 4(l)). We labeled wild type and binding sites mutant miR-145 with biotin for RNA pull down. The interaction between circCOL1A1 and miR-145 was effectively blocked by mutation, confirming the direct interaction (Figures 4(k) and 4(l)).

We next identified the level of miR-145 in gastric cancer cell lines and clinical gastric cancer tissues by qRT-PCR. The results showed that miR-145 was downregulated in gastric cancer cells and tissues, which had high levels of circCOL1A1 (Figures 4(m)–4(o)). All results reveal that circCOL1A1 regulates miR-145 by acting as a microRNA sponge.

### 3.5. RABL3 Serves as the Functional Protein of circCOL1A1/miR-145

**Figure 5:**
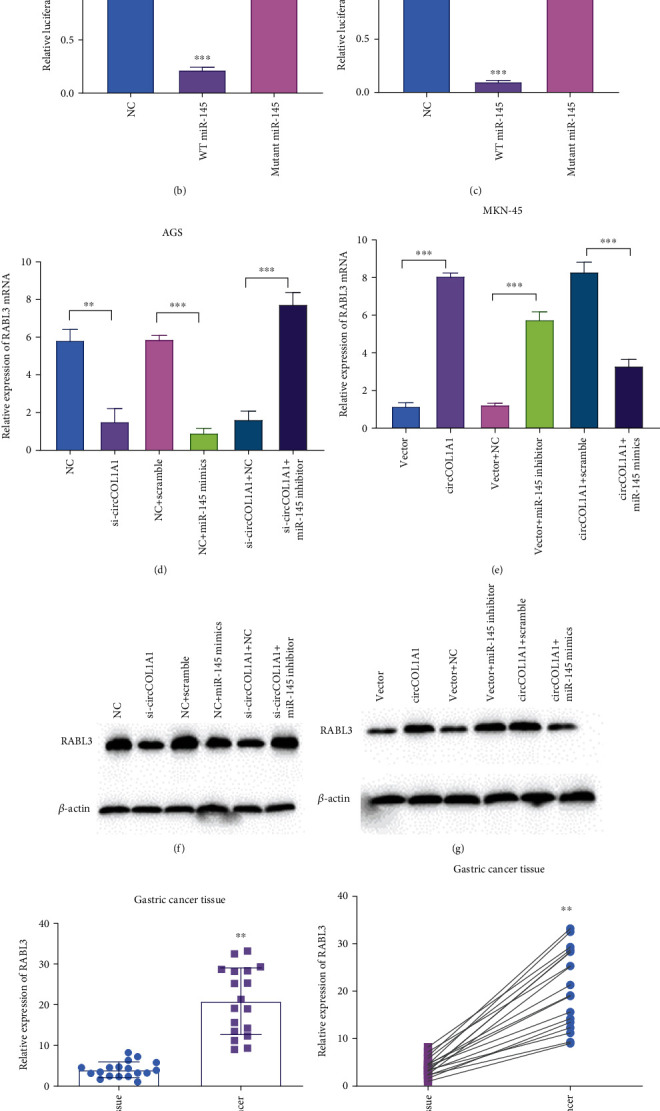
RABL3 is the target of miR-145 and regulated by circCOL1A1. (a) StarBase V3.0 predicted that RABL3 was the target gene of miR-145. The binding site was shown as indicated. (b) Luciferase reporter assays were performed in AGS transfected with wild type and mutant miR-145. (c) Luciferase reporter assays were performed in MKN-45 transfected with wild type and mutant miR-145. (d) Relative expression of RABL3 mRNA in NC and circCOL1A1 silenced AGS cells which were transfected with miR-145 mimics or inhibitors. (e) Relative expression of RABL3 mRNA in Vector and circCOL1A1 overexpressing MKN-45 cells which were transfected with miR-145 inhibitors or mimics. (f) Protein level of RABL3 was identified by western blot in AGS cell line. (g) Protein level of RABL3 was identified by western blot in the MKN-45 cell line. (h, i) RABL3 level was identified in gastric cancer tissue.

Next, we aimed to figure out the downstream protein of circCOL1A1 and miR-145. TargetScan indicated that miR-145 had complementary sites in RABL3 mRNA-3′UTR (Figure 5(a)). The luciferase activity assay showed that miR-145 mimics significantly inhibited the activity of RABL3 3′UTR, while the mutation ′effectively abrogated the influence of miR-145 on luciferase activity (Figures 5(b) and 5(c)). qRT-PCR analysis illustrated that RABL3 mRNA level decreased in circCOL1A1 silencing and miR-145 mimics overexpressing AGS cells (Figure 5(d)). The reduction of RABL3 mRNA was recovered by transfection of miR-145 inhibitor in circCOL1A1 silencing cells (Figure 5(d)). Overexpression of circCOL1A1 and miR-145 inhibitor promoted the mRNA of RABL3, and miR-145 mimics blocked the upregulation in MKN-45 (Figure 5(e)). We also applied western blot to detect the influence of circCOL1A1/miR-145 on RABL3 protein (Figures 5(f) and 5(g)). The expression levels of RABL3 were also tested in gastric cancer tissues. Results showed that circCOL1A1 was clearly upregulated clinical gastric cancer tissues (Figures 5(h) and 5(i)). These results suggest that RABL3 serves as the downstream protein of circCOL1A1/miR-145.

### 3.6. circCOL1A1/miR-145/RABL3 Contributes to the Proliferation, Migration, and Invasion of Gastric Cancer

**Figure 6:**
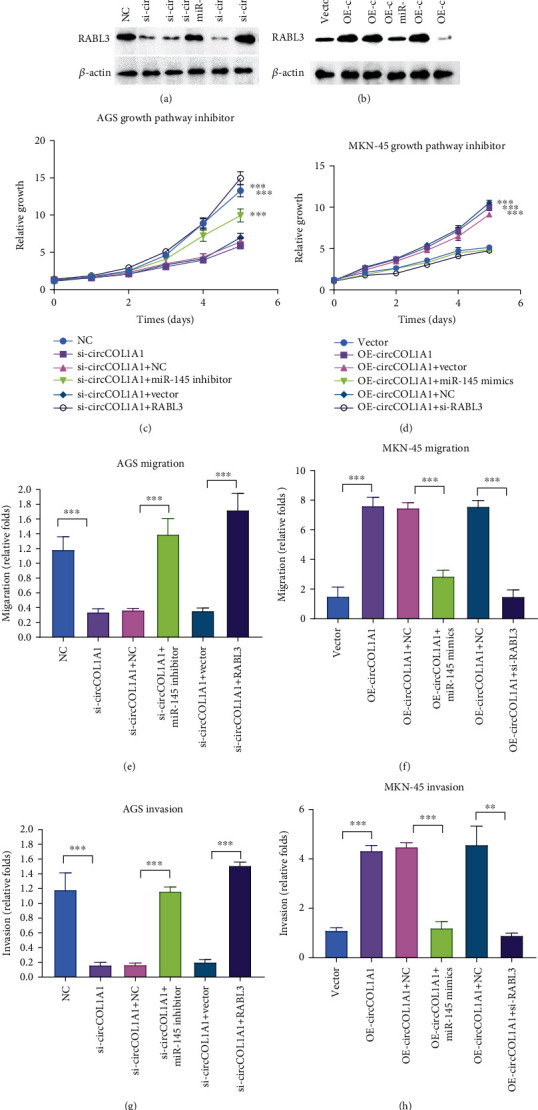
circCOL1A/miR-145/RABL3 axis regulates the proliferation, migration, and invasion of gastric cancer. (a) Western blot results of RABL3 in AGS cells. (b) Western blot results of RABL3 in MKN-45 cells. (c) CCK8 assay was used in AGS NC and si-circCOL1A1 cells. AGS NC and si-circCOL1A1 cells were rescued by transfection of the miR-145 inhibitor or RABL3 overexpression plasmids. (d) CCK8 assay was used in the MKN-45 vector and circCOL1A1 overexpression cells. MKN-45 vector and circCOL1A1 overexpression cells were then transfected with miR-145 mimics or RABL3 siRNA. (e) Migration assay was used in AGS NC and si-circCOL1A1 cells with indicated transfections. (f) Migration assay was used in the MKN-45 vector and circCOL1A1 overexpression cells with indicated transfections. (g) Invasion assay was used in AGS NC and si-circCOL1A1 cells with indicated transfections. (h) Invasion assay was used in the MKN-45 vector and circCOL1A1 overexpression cells with indicated transfections.

Whether circCOL1A1 promotes malignant progression of gastric cancer through miR-145/RABL3 axis still needs to be confirmed. We performed rescue experiments in AGS and MKN-45. CCK8 experiments showed that the silence of circCOL1A1 reduced the proliferation of AGS, which was recovered by miR-145 inhibitor or overexpression of RABL3 (Figure 6(a) and 6(c)). The promotion of proliferation induced by circCOL1A1 overexpression could be abrogated by miR-145 mimics or silence of RABL3 (Figure 6(b) and 6(d)). Meanwhile, circCOL1A1 silence-induced migration and invasion suppression could be reversed by inhibition of miR-145 or overexpression of RABL3 (Figures 6(e) and 6(g)). Additionally, miR-145 mimics or silence of RABL3 effectively blocked the enhancement of migration and invasion caused by overexpression of circCOL1A1 (Figures 6(f) and 6(h)). All the above experiments strongly showed that circCOL1A1 promoted malignancy progression by the miR-145/RABL3 pathway.

### 3.7. circCOL1A1 Promotes the Proliferation of Gastric Cancer In Vivo and the Transformation of M1/M2 Macrophage

**Figure 7:**
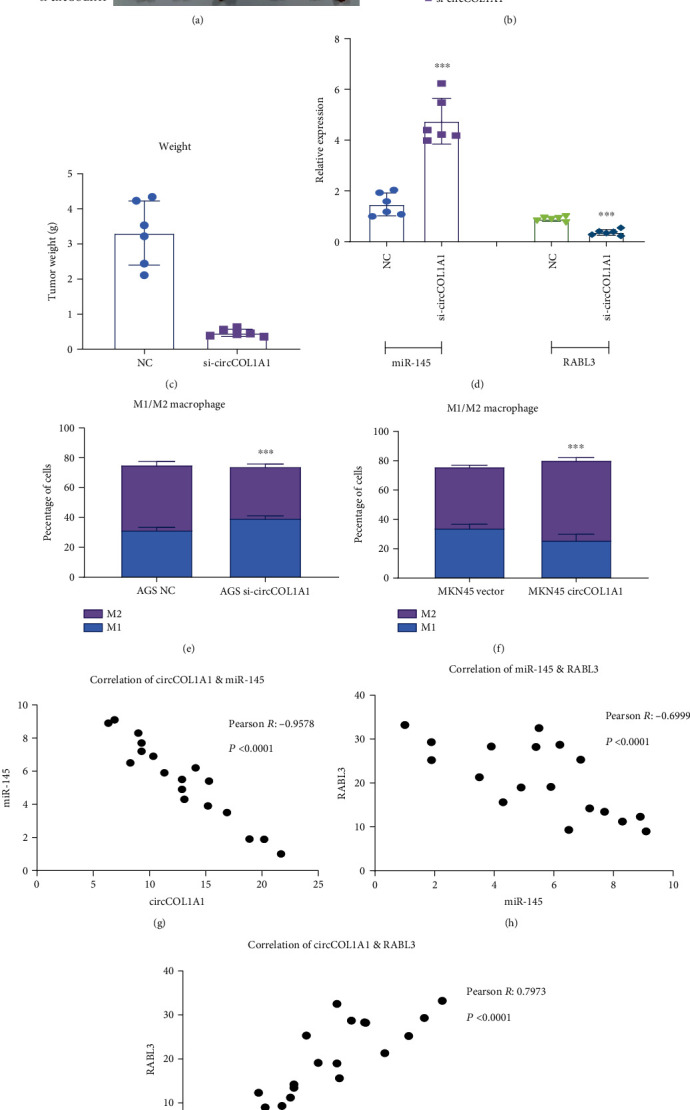
circCOL1A/miR-145/RABL3 axis in vivo. (a) 2 × 10^6^ AGS/NC and AGS/si-circCOL1A1 cells were subcutaneously injected into the ventral flanks of 5-week-old female nude mice. The tumors were measured every three days when the tumor reached 3 mm × 3 mm. At the 32th day, all mice were sacrificed, and tumors were excised and photographed. (b) The tumor volumes were calculated according to the formula: *V* = length × width^2^/2. The data indicated mean with SD of 6 tumors. (c) The weight of tumors of six tumors at the 32th day. (d) The expression of miR-145 and RABL3 in tumors of nude mice. (e) AGS was cocultured with macrophages, and flow cytometry assays were carried out to detect the percentage of M1 (CD86+) and M2 (CD206+). (f) MKN-45 was cocultured with macrophages, and flow cytometry assays were carried out to detect the percentage of M1 (CD86+) and M2 (CD206+). (g) The correlation between circCOL1A1 and miR-145. (h) The correlation between miR-145 and RABL3. (i) The correlation between circCOL1A1 and RABL3.

To confirm the in vitro findings, we then explored the effect of circCOL1A1 on tumor progression in vivo. Results showed that the silence of circCOL1A1 resulted in the decrease in the tumor growth and weight (Figures 7(a)–7(c)). Consistent with in vitro experiments, circCOL1A1 enhanced the growth of gastric cancer. We also examined the expression of circCOL1A1/miR-145/RABL3 in nude mouse tumor tissue and patient tissues. Results showed that miR-145 expression was upregulated in circCOL1A1-silenced cells, and RABL3 level decreased after silencing of circCOL1A1 (Figure 7(d)).

miR-145 is reported to play important roles in inflammatory process through multiple pathways [22], especially through the macrophage regulation. It has been reported that miR-145 enhances macrophage-mediated inflammation through targeting Arf6 [23]. miR-145 regulates the polarization of macrophage through IL-16 [24]. miR-145 also regulates macrophage immune response in tuberculosis [25]. In our research, we found that miR-145 was the downstream of circCOL1A1. We detected the influence of cirCOL1A1 on macrophage. We cocultured the macrophages with gastric cancer cells. Through flow cytometry experiments, we detected the percentage of M1 macrophage and M2 macrophage. The results showed that silencing of circCOL1A1 could decrease the percentage of M2 macrophage (Figure 7(e)). Overexpression of circCOL1A1 could enhance the transformation of M1 into M2 macrophage and increase the percentage of M2 macrophage (Figure 7(f)).

We also performed qRT-PCR in gastric cancer tissues. What is more, correlations were analyzed between circCOL1A1, miR-145, and RABL3 levels in gastric cancer tissue (Figure 7(g)–7(i)). circCOL1A1 is in a positive correlation with RABL3 and in a negative correlation with miR-145. miR-145 is also in a negative correlation with RABL3. We confirmed the existence of circCOL1A1/miR-145/RABL3 in gastric cancer tissue.

## 4. Discussion

With advances in sequencing technologies, plenty of circRNAs have been identified in various diseases [26]. Increasingly, studies suggest that circRNAs could function as tumor regulators in different mechanisms [27, 28].

In this study, we explored the existence and function of circCOL1A1 in gastric cancer. Elevated expression of circCOL1A1 (hsa_circ_0044556) was found in both gastric cancer cell lines and gastric cancer tissue. Overexpression of circCOL1A1 promotes the growth, migration, and invasion of gastric cancer. circCOL1A1 mainly exists in the cytoplasm without affecting the mRNA and protein of COL1A1. Dual-luciferase reporter assays, RNA pull-down, and qRT-PCR provided evidence that circCOL1A1 may function as a sponge for miR-145. Further experiments confirmed that RABL3 is the target gene of miR-145. Inhibition of the circCOL1A1/miR-145/RABL3 pathway effectively attenuates the proliferation, migration, and invasion of gastric cancer. These findings confirm a new tumor promoting circular RNA by regulating growth, migration, and invasion, presenting a promising target for gastric cancer therapy.

Increasing evidence suggests that circular RNAs could act as a sponge for microRNAs in diverse disease including cancer, diabetes, and cardiovascular disease [29–31]. Our results showed that circCOL1A1 mainly exists in the cytoplasm. Circular RNA Interactome (https://circinteractome.nia.nih.gov) predicted the possible targets of circCOL1A1. Real-time PCR and luciferase reporter assays confirmed that circCOL1A1 regulated the expression of miR-145 (Figure 4). Also, RNA pull-down results showed that the binding sites between miR-145 and circCOL1A1 and mutation of the binding sites abrogated the interaction between miR-145 and circCOL1A1. The results confirm that circCOL1A1 acts as a sponge for miR-145 and regulates its expression level.

MicroRNAs (miRNAs) are a family of small noncoding RNAs, which have 18-25 nucleotides. miRNAs could regulate gene expression at the transcriptional or posttranscriptional level by binding to the mRNA 3′UTR. miRNAs act as regulators in various physiological processes including proliferation, tumorigenesis, apoptosis, and drug resistance, and the abnormal level of miRNAs is widely found in diverse disease [32]. Among them, miR-145 plays crucial functions in the progression of various cancers. miR-145 is located in chromosome 5q which is a fragile region [33, 34]. Based on previous studies, miR-145 is reported to have low expression in diverse cancers, including gastric cancer, breast cancer, colorectal cancer, and osteosarcoma. Downregulation of miR-145 is reported to correlate with poor survival in gastric cancer [35]. miR-145 suppressed the malignant phenotype of gastric cancer by inhibiting expression of fascin1 [36]. miR-145 decreased the chemoresistance in gastric by targeting CD44 in gastric cancer [37]. miR-145 also regulates the invasion of gastric cancer by targeting ZEB2 and N-cadherin to regulate epithelial-mesenchymal transition [38]. It has been reported that miR-145 plays important roles in inflammatory disease. Our research focused on the regulation of miR-145. The results showed that circCOL1A1 regulated miR-145 expression through acting as a microRNA. Our research may uncover a new regulator of miR-145.

RABL3 (RAB, member of RAS oncogene family-like 3) is one member of RAB family of small GTPase which has over 70 putative members. RAB proteins could control the conversion of GDP/GTP by intrinsic GTPase activity [39, 40]. Results of protein expression in normal tissues and cell lines from ProteomicsDB, MaxQB, and MOPED show that RABL3 is expressed in the pancreas, bladder, stomach, and colon. RABL3 plays important roles in vesicular trafficking [41]. RABL3 is required for the regulation of the KRAS pathway and contributes to the cell proliferation [42]. RABL3 could regulate the prenylation of KRAS which promote cell proliferation, and RABL3 probably enhances the prenylation of other small GTPases. High level of RABL3 is related to the poor survival of non-small-cell lung cancer patients [43]. RABL3 may regulate NSCLC proliferation and migrations [44]. In our study, a high expression of miR-145 decreased both the mRNA and protein level of RABL3, and silencing of miR-145 enhanced the expression of RABL3. RABL3 is highly expressed in gastric tissues in which miR-145 is lowly expressed. The results show that miR-145 acts as a regulator of RABL3 in gastric cancer. miR-145/RABL3 might be a possible target for gastric cancer.

In our research, it is very interesting to find that circCOL1A1 increase the percentage of M2 macrophage. Macrophage polarizations are very important in the tumor immune microenvironment and promote the progression of cancer. Classically, activated M1 macrophage has a high expression of histocompatibility complex molecules; M1 macrophage is a strong killer for cancer cells. M2 macrophage exhibits anti-inflammatory and protumoral effects. M2 macrophage could promote cancer cell proliferation, invasiveness, and stemness [45]. Our study showed the influence of circCOL1A1 on macrophage M1 and M2 transformation which could provide some new thought on cirCOL1A1 function.

To our knowledge, it is the first research about circCOL1A. This study investigates the existence, expression, and function of cirCOL1A1 in gastric cancer. What is more, it is also the first study to investigate the relationship between miR-145 and RABL3. Also, the regulation of circCOL1A1/miR-145/RABL3 could influence the proliferation, migration, invasion, and the transformation of M1 into M2 macrophage.

Given the previous results, we considered that the circCOL1A1/miR-145/RABL3 axis might be involved in the progress of gastric cancer. These findings might bring some new thoughts for the treatment of gastric cancer.

## 5. Conclusion

In summary, our study uncovered the oncogenic effects of circCOL1A1 on gastric cancer. circCOL1A1 could promote proliferation, migration, invasion, and the transformation of M1 into M2 macrophage.

## Figures and Tables

**Table 1:** 

	Left primer	Right primer
circCOL1A1	GAAGCTGGTCTGCCTGGTG	GAGGAGCGAAAGGAAGGAGA
Linear circCOL1A1	GAGGGCCAAGACGAAGACAT	CAGATCACGTCATCGCACAAC
RABL3	TCCCTGGATCGGGTGAAGG	GCACTTGATTTTGGCATAGGAGA

## Data Availability

The data that support the findings of this study are available from the corresponding author upon reasonable request.
